# Hip Abduction Can Prevent Posterior Edge Loading of Hip Replacements

**DOI:** 10.1002/jor.22364

**Published:** 2013-04-10

**Authors:** Richard J van Arkel, Luca Modenese, Andrew TM Phillips, Jonathan RT Jeffers

**Affiliations:** 1 Medical Engineering, Department of Mechanical Engineering, Imperial College LondonLondon, SW7 2AZ, United Kingdom; 2 Structural Biomechanics, Department of Civil and Environmental Engineering, Imperial College LondonLondon, United Kingdom; 3 Centre for Musculoskeletal Research, Griffith Health Institute, Griffith University, Gold CoastQueensland, Australia

**Keywords:** avoid edge loading, muscles, hip, ceramic-on-ceramic, metal-on-metal

## Abstract

Edge loading causes clinical problems for hard-on-hard hip replacements, and edge loading wear scars are present on the majority of retrieved components. We asked the question: are the lines of action of hip joint muscles such that edge loading can occur in a well-designed, well-positioned acetabular cup? A musculoskeletal model, based on cadaveric lower limb geometry, was used to calculate for each muscle, in every position within the complete range of motion, whether its contraction would safely pull the femoral head into the cup or contribute to edge loading. The results show that all the muscles that insert into the distal femur, patella, or tibia could cause edge loading of a well-positioned cup when the hip is in deep flexion. Patients frequently use distally inserting muscles for movements requiring deep hip flexion, such as sit-to-stand. Importantly, the results, which are supported by in vivo data and clinical findings, also show that risk of edge loading is dramatically reduced by combining deep hip flexion with hip abduction. Patients, including those with sub-optimally positioned cups, may be able to reduce the prevalence of edge loading by rising from chairs or stooping with the hip abducted. © 2013 Orthopaedic Research Society Published by Wiley Periodicals, Inc. J Orthop Res 31:1172–1179, 2013.

Edge loading damages hard-on-hard hip replacements and causes clinical problems: for metal-on-metal (MoM) implants, excessive edge loading wear can lead to pseudotumors and early revision^1^ and for ceramic-on-ceramic (CoC) bearings, edge loading has been related to higher wear rates and audible hip joint squeaking.^2^ Edge loading describes the increased contact stress resulting from a decreased contact area between the acetabular cup and femoral head at the rim of the cup. It occurs when the cup provides insufficient coverage of the head preventing a full circular contact area from developing around the hip joint contact force vector.^3^ This mechanism particularly affects MoM implants with reduced cup subtended angles^3^ and/or poor cup positioning,^4^ as these factors bring the rim closer to the path of the contact vector and expose the hip to edge loading.^5^ However, clinical evidence also exists of unexplained edge loading wear on retrievals from well-designed, well-positioned MoM components^3^ and a recent in vivo MoM resurfacing study showed that posterior edge loading occurs in all hips in all patients when extending from deep hip flexion when rising from a chair.^5^

Edge loading can also occur as a consequence of near-dislocation events: anterior impingement in deep hip flexion and internal rotation, the most common mechanism, causes small subluxations of the head that exposes it to posterior edge loading on the hard edge of the cup, leading to extreme contact stresses and wear.^6^ However CoC implant retrievals showed that posterior edge loading wear scars are present on the majority of bearings and occur most commonly in the absence of impingement.^7^,^8^

Given the high incidence of posterior edge loading reported in the absence of impingement and the strong influence of muscles on the hip joint contact force,^9^ we hypothesised that the lines of action of muscles are such that edge loading can occur in all hips when they are deeply flexed during routine activities, and so we addressed two research questions: are the lines of action of hip joint muscles such that they could cause edge loading of a well-designed, well-positioned acetabular cup?; and, how sensitive are the results to geometrical variation of the cup through changes to the implant design or orientation?

## METHODS

### Muscle Contribution to Edge Loading

A lower limb model was developed based on a digitized cadaveric right leg specimen that detailed muscle origin and insertion points.^10^ More detailed information about the model can be found in a previous study that compared computed hip joint contact force magnitudes with those measured in vivo.^11^ The model's muscle geometry included an anatomical wrap for the iliopsoas muscle fibers around the pelvis to ensure it pulled the femur in the correct direction. To keep representative muscle geometry throughout a complete range of motion, additional muscle wrapping surfaces were applied to the gluteus maximus superior fibers, gluteus maximus inferior fibers, the gemelli, and obturator internus (see Appendix A).

The full hip range of motion for an adult male^12^ was discretized into 5° positions of flexion (−10° to 120°), abduction (−25° to 40°), and rotation (−40° to 40°) totaling 6,426 hip orientations. Angles were referenced in accordance with the ISB recommendations for joint coordinate systems.^13^ OpenSim version 2.4.0^14^ was used to place the model in each of these static positions, and the direction of the force vector exerted by each muscle onto the pelvis was calculated using an OpenSim plugin, which is available for free download together with detailed documentation.^15^ An overview of how the plugin works is included in Appendix B.

**Figure 1:**
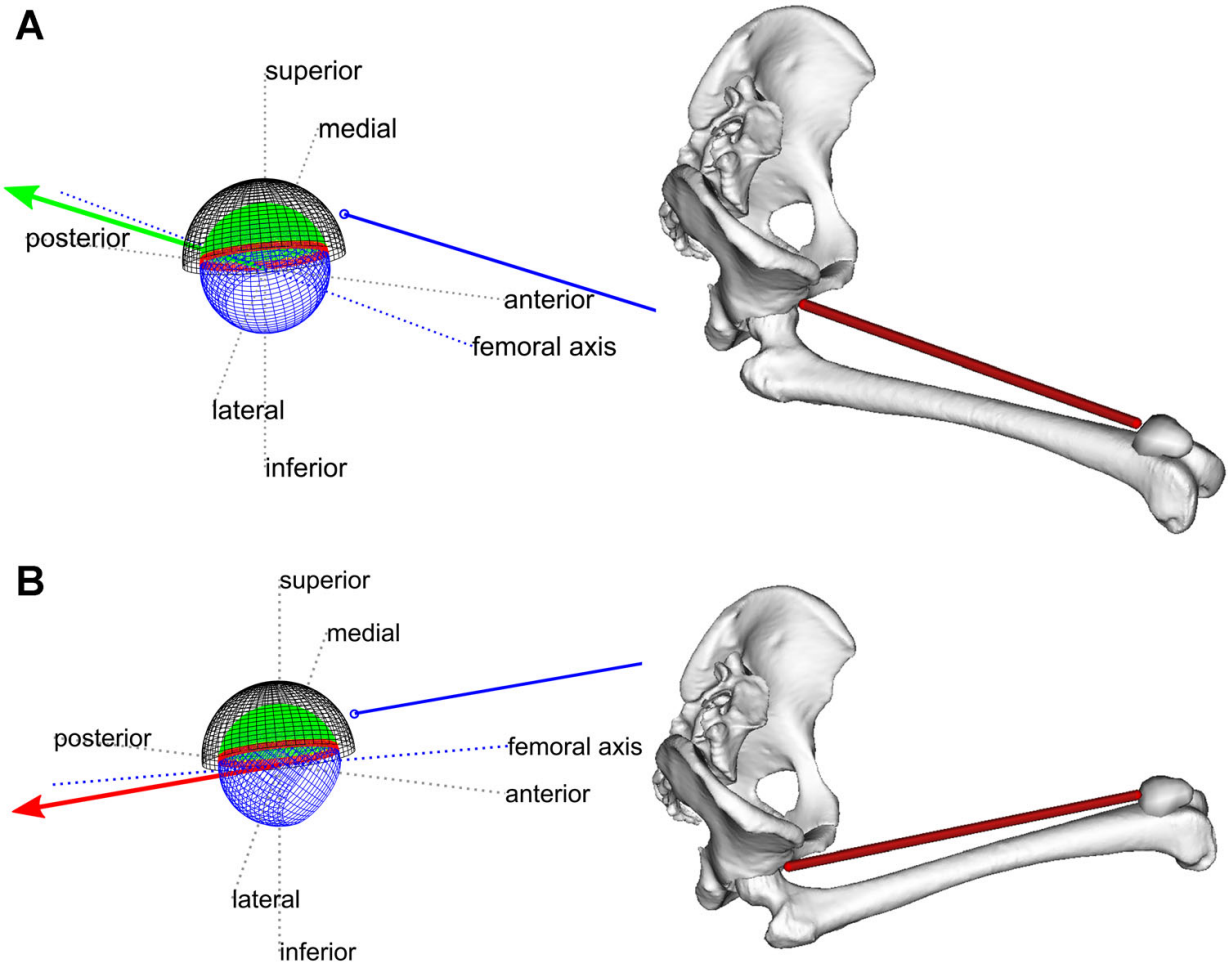
(A and B) Diagrams of the line of action of the rectus femoris (blue line) and its unit reaction force at the hip joint (red/green arrow) at 90° flexion and neutral rotation. The cup liner is divided into a green safe zone and a red edge load risk-zone. (A) The hip is abducted 20° and the rectus femoris pulls the head (blue sphere) into the cup safe zone. (B) The hip is adducted 20°, now the line of action of the rectus femoris pulls the head out of the cup and thus it could contribute to an edge loading contact vector. Representative images from the musculoskeletal model are shown.

The hip was modeled in MatLab (version 2011b, The MathWorks, Inc., Austin, TX) as a typical Ø28 mm bearing with an acetabular subtended arc angle of 168° (e.g., the Biolox Forte cup) well-positioned at 20° anteversion and 45° inclination using the radiographic definition.^16^ A conservative edge loading risk-zone, which allows for the circular contact patch surrounding the force vector, was defined within 5° of the cup edge. The unit force vectors acting on the pelvis calculated by the plugin were applied at the center of rotation as equal and opposite unit reaction forces. For each muscle, we calculated if its contraction would safely pull the head into the cup (Fig. 1A) or contribute towards creating an edge loading force vector (Fig. 1B).

### Effects of Implant Design

The muscle contribution to edge loading was then re-calculated for a well-positioned cup for the implant designs in Table 1. The effect of varying the edge load risk-zone was studied by varying the angle in the range 5–30°, which represents the range of possible contact patch semi-angles for CoC^17^ and MoM bearings.^5^,^18^

**Table 1:** The Models and Dimensions of Implant Designs Studied

Material Couple	Implant	Head Diameter (mm)	Subtended Angle (°)
CoC	Biolox Forte	28	168
CoC	Delta motion	36	168
MoM	Adept	38	161
MoM	Adept	58	161
MoM	ASR	59	153
MoM	ASR	39	144

### Effects of Implant Orientation

For the original cup design, the effects of different acetabular orientations were investigated by varying the angles for all nine possible combinations of 5°, 20°, and 35° (low, medium, and high) anteversion with 30°, 45°, and 60° (low, medium, and high) inclination.

### Comparison with In Vivo Force Data

**Figure 2:**
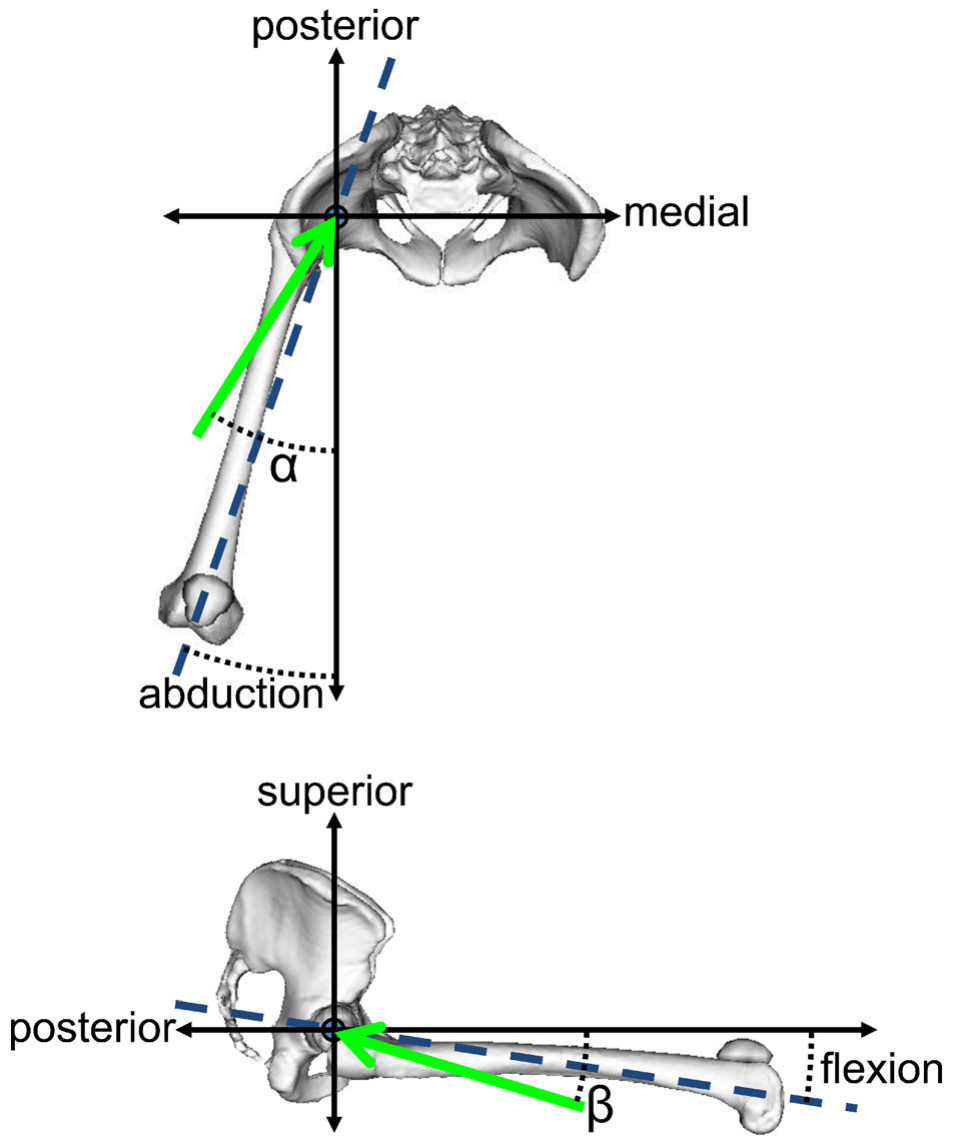
The definitions of *α* and *β* in the transverse and sagittal planes, respectively; the green arrows are projections of the resultant force at the pelvis into these planes, and the blue dashed lines highlight the femoral axis.

Bergmann's in vivo tests^9^ provide kinematic and force data for 16 trials of sit-to-stand. The data were retrieved from HIP98^9^ and used to test the correlation between hip flexion angle, abduction and rotation at the point of maximum hip joint contact force, and two angles that define how much the force points into the cup: *α* in the transverse plane, and *β* in the sagittal plane (Fig. 2). For 15 of the trials, the maximum hip contact force occurs at, or shortly after the point of seat off and maximum hip flexion; however, the trial HSRCU3 has unique dynamics and is less suitable to study forces in deep flexion because the maximum load occurs much later than the point of seat off. Thus, the data were tested both with and without trial HSRCU3. A 5° knee varus angle, which does not affect correlation statistics, was used to convert from Bergmann's *z*-axis^9^ to the ISB's *y*-axis.^13^

## RESULTS

### Muscle Contribution to Edge Loading

**Figure 3:**
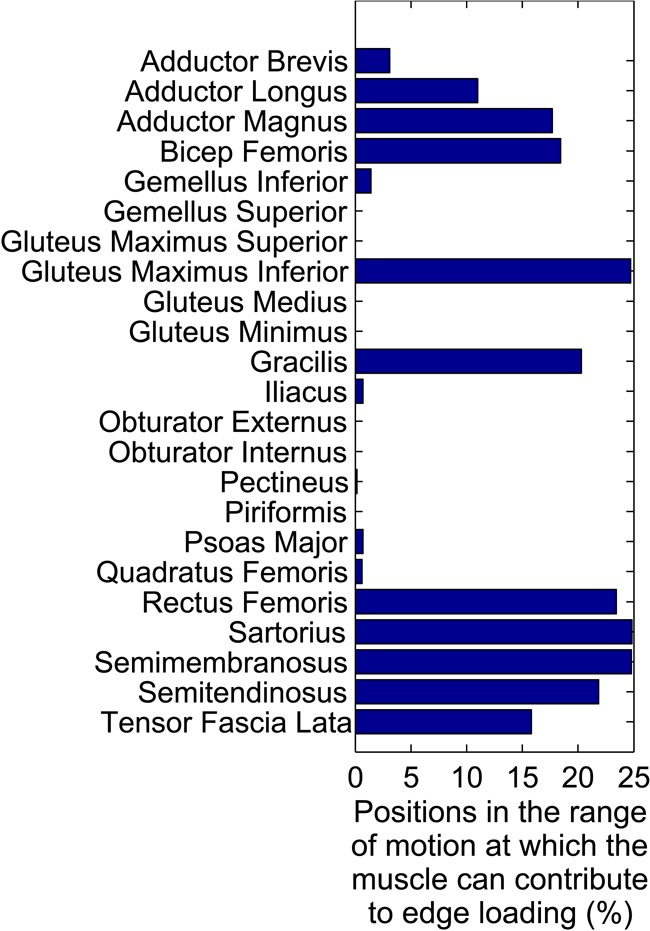
List of the muscles included in the study indicating the percentage of positions in the complete range of motion at which each muscle could contribute to an edge loading force vector in a well-positioned cup.

All the muscles that inserted into the distal femur, patella, or tibia can contribute to edge loading of a well-positioned cup within a normal range of motion, whereas other large muscles, such as the gluteus medius, cannot. Figure 3 lists the included muscles and the percentage of positions in the range of motion where the line of action of that muscle could contribute to an edge loading hip contact force.

**Figure 4:**
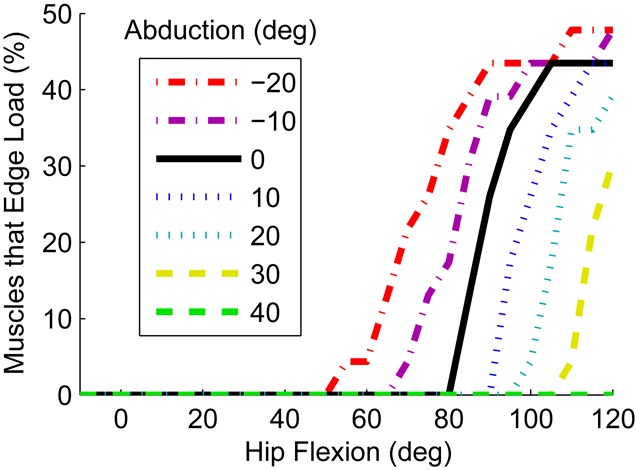
The percentage of muscles that can contribute to edge loading as a function of hip flexion with neutral rotation and different ab/adduction in a well-positioned cup.

The risk of edge loading was particularly prevalent during deep flexion (Fig. 4). For a well-positioned cup, the percentage of muscles that could contribute to edge loading increased from 0% to 39% (9/23) as flexion increased from 80° to 100° with neutral abduction and rotation.

Hip abduction dramatically reduced the muscular contribution to edge loading in deep flexion (Fig. 4); at ≥20° abduction, no muscles contributed to edge loading up to 95° of flexion. Hip flexion with adduction had the opposite effect; when the hip was in 20° adduction, muscles could cause edge loading above 50° flexion. Internal or external rotation of the hip made little difference to the risk of edge loading.

### Effects of Implant Design

**Figure 5:**
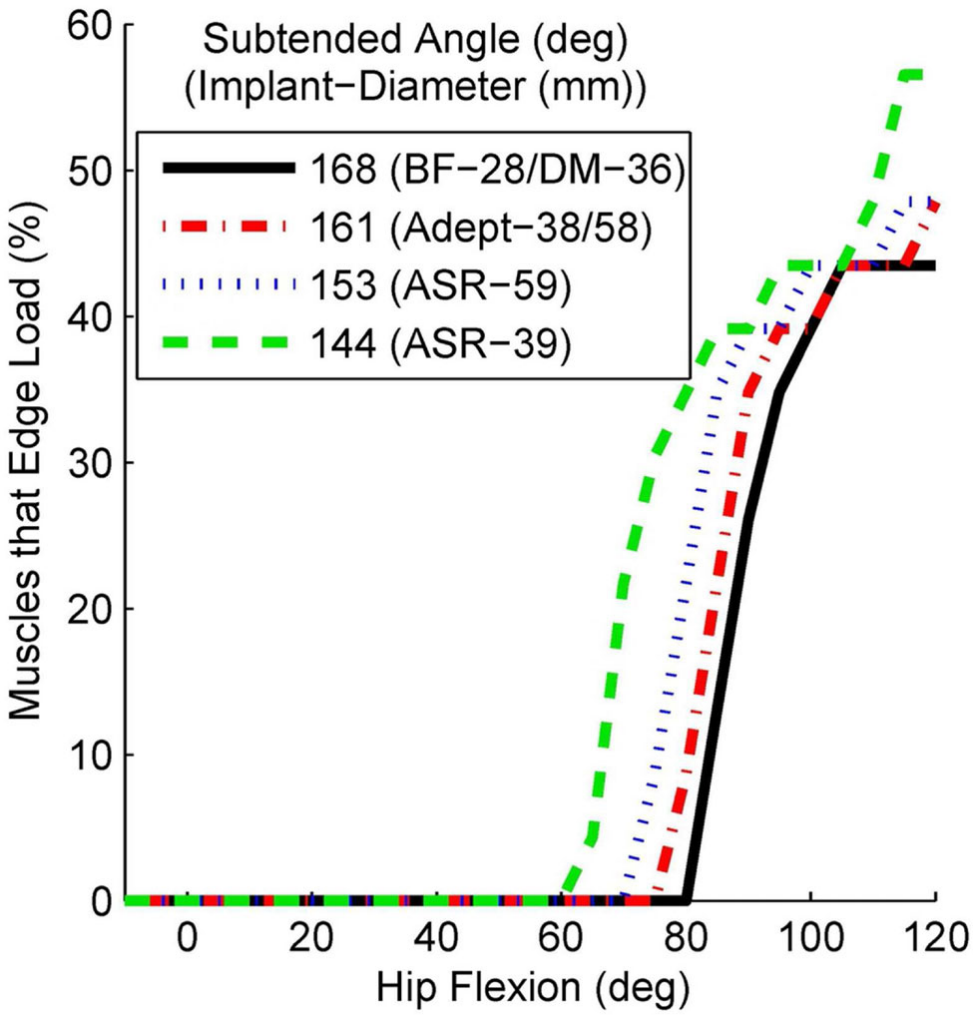
The effect of reducing the subtended angle of the cup arc on the possible muscle contribution to edge loading for a well-positioned cup with neutral hip abduction and rotation.

Decreasing the subtended angle of the cup arc increased the maximum possible muscle contribution to edge loading and decreased the flexion angle at which muscle contribution to edge loading was possible (Fig. 5). However, changing the size of the bearing in isolation did not affect the possible muscular contribution to edge loading. Changing the edge load risk-zone had the same effect as decreasing the subtended angle as both changes reduced the safe coverage of the head. For example, two bearings with subtended angles of 168° and 152° and edge load risk-zones of 13° and 5°, respectively, were equivalent (safe coverage arcs of 142°).

### Effects of Implant Orientation

For all cup positions, the general trend was the same as shown in Figure 4; the percentage of muscles that can contribute to edge loading increased rapidly at a given flexion angle, was highest in deep flexion, and abducting the hip had a protective function. Internal and external rotation had a larger effect in some cup positions in comparison to a well-positioned cup; however, the dominant effect was still driven by hip flexion, then ab/adduction.

**Figure 6:**
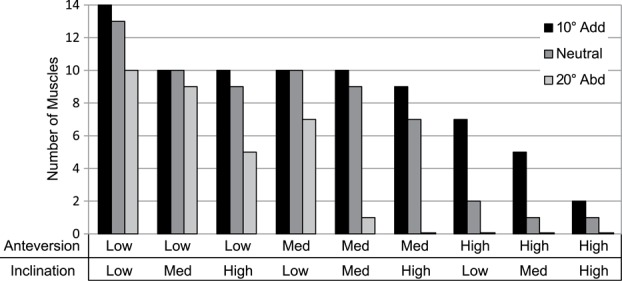
The number of muscles that can contribute to edge loading of a well-designed cup at 100° hip flexion and neutral hip rotation with varying hip abduction and cup orientation.

The following trends are based on data from the complete range of motion; however, many can be seen in Figure 6. Low anteversion decreased the flexion angle at which edge loading could occur but had little effect on the maximum number of muscles that could edge load a hip; it effectively shifted the lines in Figure 4 to the left. High anteversion had the opposite effect; it allowed higher flexion angles before large numbers of distal muscles could contribute to edge loading.

Low inclination had two effects: it increased the maximum number of muscles that can cause edge loading forces over all flexion angles, because some of the short external rotators and obturator muscles had contact vectors that were in the inferior portion of the risk zone. It also reduced, but did not eliminate, the effect of abducting the hip in deep flexion on the number of muscles that can contribute edge loading force components.

High inclination had three effects. First, it decreased the number of distally inserting muscles that can contribute to edge loading in flexion. Second, it increased the effect of abducting the hip during flexion. Third, it allowed the iliopsoas muscles to contribute to edge loading forces in low flexion or extension angles, and also the distally inserting muscles when the hip was adducted in low flexion or extension.

Combining high/low anteversion with high/low inclination provided a combination of the above effects. For example low inclination and low anteversion resulted in high muscle contribution to edge loading at lower flexion angles.

### Comparison with In Vivo Force Data

**Figure 7:**
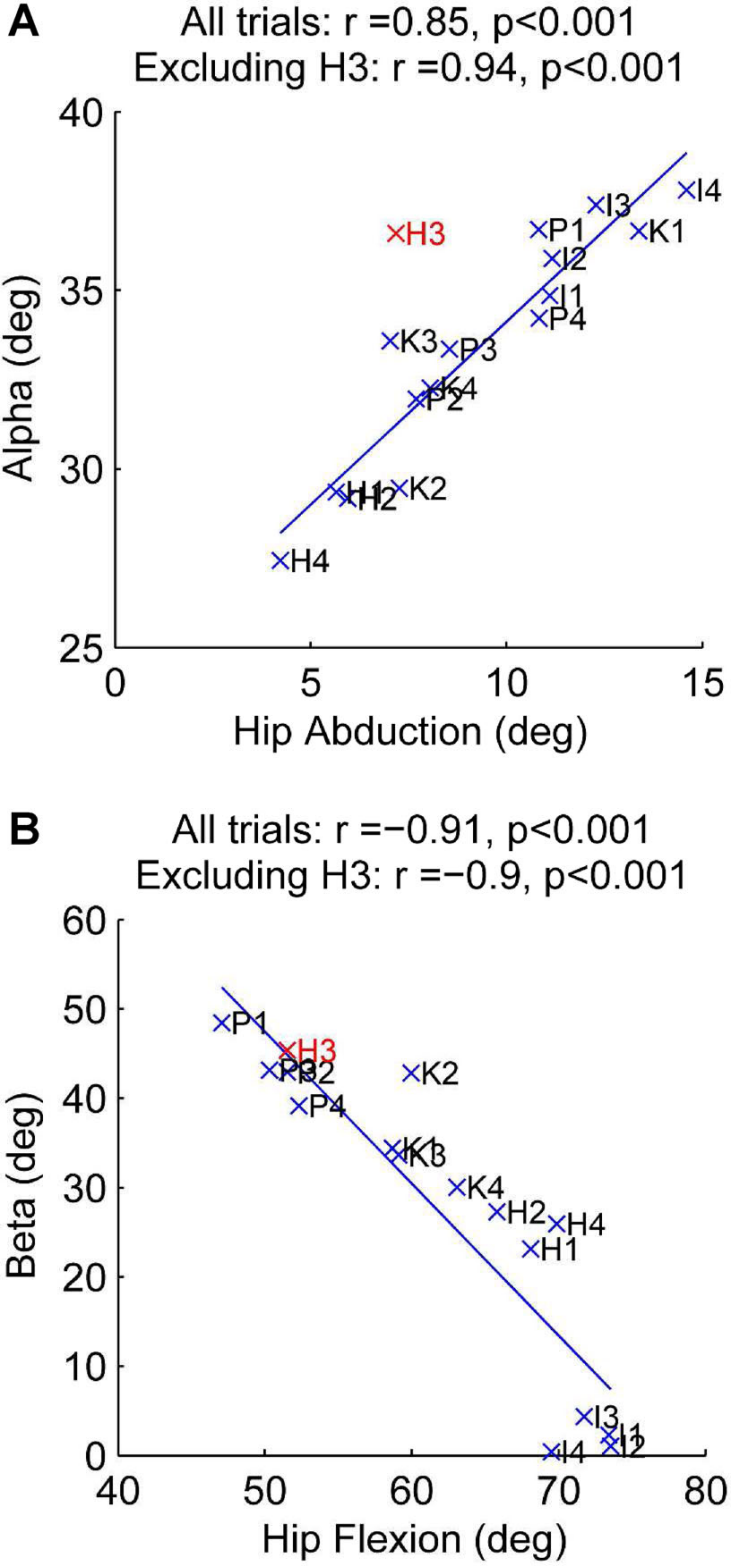
The correlation between the direction of the contact vector relative to the pelvis and the position of the hip. Points are labeled with the first letter of the trial name and trial number according to the sit-to-stand trial in HIP98 (e.g., H1 = HSRCU1 in HIP98), and lines of best fit are shown. The trial with abnormal dynamics (HSRCU3) is highlighted in red.

At maximum load, strong, significant correlations existed between the abduction angle and *α* (Fig. 7a, *r* = 0.85, *p*-value < 0.001), and the flexion angle and *β* (Figure 7b, *r* = −0.91, *p*-value < 0.001). Excluding the abnormal trial HSRCU3 resulted in an even stronger correlation between abduction and *α* (Fig. 7a, *r* = 0.94, *p*-value < 0.001).

## DISCUSSION

We showed that the lines of action of distally inserting muscles can contribute to edge loading of a well-positioned acetabular cup when the hip is in deep flexion, and abducting the flexed hip moves the lines of action of these muscles away from the edge and into a safe-zone inside the cup. This is because the lines of action of the distally inserting muscles are tied to the position of the femur (Fig. 1). The positive benefit of abduction is true for all cup designs (Table 1) and orientations tested (Fig. 6). Incorporating abduction into activities in deep flexion, like sit-to-stand, may be a useful rehabilitation exercise for patients to avoid edge loading wear, and this may be particularly beneficial to patients with the ASR implant. Moreover, combining high flexion angles with abduction could also prevent shear dislocation (without impingement)^19^,^20^ by bringing the lines of action of all the muscles to within the cup (Fig. 4). Abduction of a flexed hip also moves the femoral neck and surrounding bone away from the anterior portion of the acetabulum and pelvis, the most common deep flexion impingement site,^20^,^21^ adding further weight to the finding that hip abduction in deep hip flexion is of benefit to patients.

The adopted methodology is purely geometrical and does not include explicit calculation of the hip contact force vector because the individual muscle contributions are assessed only with respect to their direction. Despite this limitation, the data showed strong equivalence to the resultant load vector measured in instrumented implants. Also, while our study cannot find specific hip positions or movements that cause edge loading, it does show that the lines of action of muscles are such that edge loading of a well-positioned cup in the absence of subluxation is only possible in deep flexion, and abducting the hip can prevent this. Indeed, the approach avoids some of the limitations associated with determining force magnitudes through modeling: first, it does not require an optimization routine. A recent study showed that a musculoskeletal model based on the same anatomic dataset used here could potentially reproduce the hip contact force direction measured in vivo, but the optimization techniques currently employed for estimating muscle forces are unable to yield muscle recruitment adequate to accurately estimate that vector.^22^ Second, it allows the full range of motion to be explored, and so the results encompass all the activities that a hip replacement patient could do.

In vivo resultant joint reaction force measurements from instrumented implants^9^ corroborate the findings by showing that the direction of the maximum resultant joint force relative to the pelvis is highly correlated with the position of the femur during sit-to-stand activity (Fig. 7). The in vivo data show that posterior edge loading is possible in deep flexion as flexion is correlated with a more posteriorly pointing load vector (Fig. 7B). It also supports the result that activity modification can reduce the risk of edge loading: higher abduction at the point of seat off was strongly correlated with a more medially angled force relative to the pelvis, and hence a force that points more inbound, further away from the posterior edge of the acetabulum (Fig. 7A).

Rising from a chair can require >100° of hip flexion, with abduction varying from −10° to 20°.^9^,^19^ This movement relies on considerable muscle force from the distally inserting hamstrings, rectus femoris, and gluteus maximus, and little contribution from the gluteus medius and short external rotators.^20^,^23^,^24^ Hence, the muscles that can contribute to creating an edge loading force (Fig. 3) during deep flexion (Fig. 4) are known to be highly active during sit-to-stand, while muscles that provide a protective function are not. This may explain the high incidence of edge loading wear reported clinically,^1^,^3^,^4^,^8^ and why edge loading occurred in all MoM resurfacing patients when rising from a chair.^5^

The implant sensitivity study showed that decreasing the subtended cup angle increased the possibility of muscle contribution to edge loading (Fig. 5). This supports results from explanted MoM bearings that show that cups with reduced subtended angles edge loaded significantly more and suffered significantly higher wear rates^3^ and emphasizes the need to mitigate the risks of edge loading for new cup designs that have reduced subtended arcs.

Our results support findings from ceramic retrievals where the majority of edge loading wear occurred posteriorly during high flexion^7^,^8^ with low cup anteversion increasing the risk.^25^ Interestingly, we also showed that high inclination can help protect against posterior edge loading by moving the inferior edge of the cup more laterally and thus in an anteverted cup it provides more posterior coverage of the head. However, high inclination should be avoided as it can expose the joint to superior edge loading in low flexion or extension angles, and edge loading during gait can have severe consequences.^4^,^5^,^8^ Indeed recent MoM resurfacing research using AP X-rays suggests that low inclination is beneficial, particularly for small bearings.^26^ However, in both established^8^ and contemporary^27^ CoC bearings, a combination of low inclination and low anteversion led to high incidences of posterior edge loading wear and squeaking. This is the cup orientation at greatest risk of posterior edge loading from muscle action (Fig. 6), and so low inclination should be combined with higher anteversion to provide better coverage of the head throughout the range of motion.

Muscles damaged in the most common surgical approaches (lateral: gluteus medius and minimus, posterior: short external rotators)^28^ never cause edge loading of well-positioned cups (Fig. 3). Intraoperative repair and rehabilitative strengthening of these muscles may reduce the risk of edge loading as weakened muscles may lead to the patient substituting their function for a distally inserting alternative^29^ that could contribute to an edge loading force.

Edge loading is caused by soft tissue laxity leading to microseparation during gait,^30^ by impingement in deep flexion with internal rotation,^6^ or by low subtended angles and/or high inclination providing insufficient superior coverage of the head.^4^,^5^ We do not discount these phenomena but provide an additional mechanism by which edge loading could occur in all hip replacement patients: posteriorly in deep flexion due to muscle forces alone.

In answer to our research questions, we showed that all the distally inserting muscles could cause edge loading of well-designed, well-positioned acetabular cups when the hip is deeply flexed. Low subtended arc angles and suboptimal cup orientation can increase the risk of edge loading through muscle action, but does not alter the general trend observed for a well-designed, well-positioned cup. However, our most important finding is that all patients, regardless of how their prosthesis was designed or implanted, can reduce the prevalence of posterior edge loading, and perhaps dislocation, by introducing abduction to activities that require deep flexion; this can easily be implemented for activities such as rising from a chair and stooping by separating the knees before performing the movement.

## Figures and Tables

**Figure A1:**
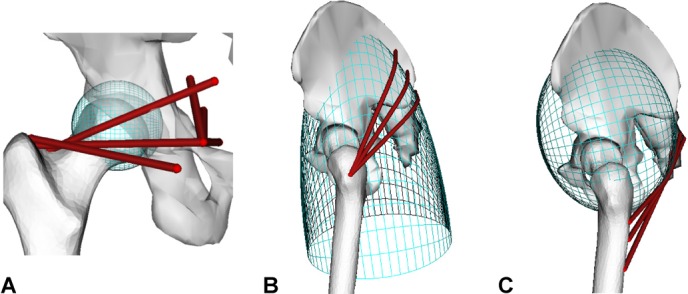
***Wrapping surfaces included in the model in order to improve the muscle geometrical representation (A) of the muscles surrounding the femoral head (the *gemelli* and *obturator internus*), (B) of the superior bundles of the *gluteus maximus* and (C) of the inferior bundles of the *gluteus maximus*.

## APPENDIX A

### A.1 Muscle wrapping geometries

The musculoskeletal model used in this investigation is based on the anatomical measurements collected by Klein Horsman et al.^10^ from a single cadaveric specimen. The original dataset has been enhanced at the hip joint by including the following wrapping surfaces:

1. The hip joint capsule was represented as a sphere centered in the hip joint center (Fig. 8). This modeling choice is consistent with the previous investigation of Brand et al.^31^ and prevents muscle fibres of the *gemelli* and the *obturator internus* from crossing the femoral head at high hip flexion angles.
2. As medical images for the specimen dissected by Klein Horsman et al.^10^ were not made available, the anatomical dataset released through the Living Human Digital Library project (LHDL),^32^ and publicly available at https://www.physiomespace.com was used to redesign the *gluteus maximus* geometry. As this dataset makes available muscle fiber paths collected on the muscle surface, an ellipsoid was fitted in a least squares sense to the point cloud obtained from the *gluteus maximus* fibers. In order to take into account the flattening of the muscle due to the supine position of the specimen, the ellipsoid axes were varied under the constraint of constant volume and finally scaled to the dimensions of the Klein Horsman specimen using a scaling ratio based on the thigh length. The obtained surface was used for defining a wrapping surface for the upper bundles of *gluteus maximus* (Fig. 8B).
3. An additional wrapping surface representing the ischial tuberosity was included in the model in order to influence the paths of the *gluteus maximus* inferior bundles (Fig. 8C). A similar modeling choice can be found in a previously published model of the lower limb.^33^,^34^ Due to the difference in wraps for the superior and inferior fiber bundles of the *gluteus maximus*, it is reported in the main text as two different muscles to indicate how the different fiber bundles would contribute to the hip joint reaction force.

## APPENDIX B

### B.1 Plugin overview

The MuscleForceDirection plugin executes a few simple operations. Given a selected body (or a set of bodies) included in an OpenSim model, the plugin:

1. Identifies the muscles attached to the segment(s).
2. Retrieves the current path for each muscle, including wrapping points, by using the GetPointForceDirections method of the class OpenSim::GeometryPath.
3. Identifies the anatomical or effective muscle attachments according to the user selection and calculates the muscle force direction at that point.
4. Depending on the reference system chosen by the user (body reference system or global coordinate system), the plugin transforms the previously identified muscle attachment coordinates and force directions by using the methods of the OpenSim::SimbodyEngine class.
5. Prints the muscle force directions and, if requested, the muscle attachments.

### B.2 More information

Full documentation and the plugin can be downloaded for free from https://simtk.org/home/force_direction.^15^
